# Kappa opioid regulation of depressive-like behavior during acute withdrawal and protracted abstinence from ethanol

**DOI:** 10.1371/journal.pone.0205016

**Published:** 2018-09-28

**Authors:** Sorscha K. Jarman, Alison M. Haney, Glenn R. Valdez

**Affiliations:** Department of Psychology, Grand Valley State University, Allendale, MI, United States of America; Radboud University Medical Centre, NETHERLANDS

## Abstract

The dynorphin/kappa opioid receptor (DYN/KOR) system appears to be a key mediator of the behavioral effects of chronic exposure to alcohol. Although KOR opioid receptor antagonists have been shown to decrease stress-related behaviors in animal models during acute ethanol withdrawal, the role of the DYN/KOR system in regulating long-term behavioral changes following protracted abstinence from ethanol is not well understood. The objective of the current study was to further explore the role of the DYN/KOR system in regulating stress-related behavioral changes associated with acute withdrawal and protracted abstinence from ethanol. More specifically, the present experiments sought to examine the ability of the KOR antagonist norbinaltorphimine (nor-BNI) to reverse depressive-like behavior in the forced swim test in rats exposed to chronic ethanol. In the first experiment, rats were fed an ethanol or control liquid diet for 28–30 days, and then 24 hours after removal of the diet, were exposed to inescapable swim stress. Immediately following this procedure, rats were injected with nor-BNI (20 mg/kg) or saline and then tested 24 hours later in the forced swim test. A second experiment used a similar procedure to examine the effects of nor-BNI on behavioral changes during protracted abstinence in rats tested in the forced swim test 3 weeks after exposure to the ethanol liquid diet procedure. Ethanol-dependent rats showed increased immobility, which is thought to indicate a depressive-like state, when examined during acute withdrawal and protracted abstinence compared to controls, an effect attenuated by nor-BNI. These results suggest that the DYN/KOR system plays role in mediating both short- and long-term behavioral changes associated with depression following chronic alcohol exposure.

## Introduction

During withdrawal, individuals with Alcohol Use Disorder often experience negative mood states such as depression, which clinical studies suggest are among the most common reasons for relapse [1–4]. The chronic nature of this negative affect poses a significant challenge to relapse prevention [5]. These long-term changes have also been observed in preclinical studies, as laboratory animals exposed to chronic ethanol display long-term behavioral changes in models of anxiety [6–9], depression [10, 11], and relapse [7, 12, 13]. The correlation between changes in stress-related behaviors in these models and increased ethanol self-administration long after chronic ethanol exposure suggests that the underlying negative affective-like states are a significant contributing factor to relapse.

The neurobiological mechanisms mediating the relationship between negative affect and relapse are still not completely understood. One system that may play a significant role is the dynorphin (DYN)/kappa opioid receptor (KOR) system, which has been previously described as a key regulator of alcohol-related stress [14]. During acute withdrawal, the KOR antagonists norbinaltorphimine (nor-BNI) [15] and JDTic [16] reduce stress-related behaviors in the elevated plus maze, an animal model of anxiety, and nor-BNI attenuates heightened ethanol self-administration [17–19]. Mice exposed to chronic intermittent ethanol also show increased ethanol consumption following exposure to inescapable swim stress or injection of the KOR agonist U50,488, an effect reversed by the KOR antagonist LY2444296 [20]. nor-BNI also attenuates U50,488-induced reinstatement of ethanol seeking in rats trained to self-administer ethanol [21]. Although these studies suggest that the DYN/KOR system is involved in regulating the acute effects of ethanol withdrawal and exposure, the role of KORs in mediating long-term changes due to protracted periods of abstinence has yet to be fully explored.

Depressive symptoms appear to be a key contributor in stress-related relapse [3, 22], and chronic ethanol exposure leads to long-term behavioral changes in animal models of depression [10, 11]. Similar to what has been observed during acute withdrawal, the DYN/KOR system may also be involved in regulating the long-term behavioral changes associated with chronic ethanol. For example, nor-BNI reduces the enhanced responsiveness to stress seen in rats tested in the elevated plus maze with a history of ethanol dependence following 6 weeks of abstinence [9]. Injections of nor-BNI into the amygdala also attenuate increases in ethanol self-administration in rats exposed to chronic ethanol vapor 30 days post withdrawal without affecting physical withdrawal signs [13].

A more complete characterization of the neurobiological mechanisms underlying the long-term behavioral changes associated with chronic ethanol exposure may lead to a greater understanding of stress-related relapse. To this end, the present study will further explore the role of the DYN/KOR system in regulating depressive-like behaviors associated with long-term withdrawal from ethanol. Specifically, the current experiments examine the ability of nor-BNI to reverse increases in depressive-like behavior observed in the forced swim test during acute withdrawal and protracted abstinence from ethanol.

## Methods

### Animals

Male Wistar rats (Charles River, Portage, MI; n = 68) were used in this experiment. Body weights were 200–250 g and age was approximately 60 days at the start of the experiments. Rats were group housed (2–3 per cage) with food and water available ad libitum, except during liquid diet administration, and were weighed daily. Rats were maintained on a 12-hour light/dark cycle (lights on at 22:00 h). Procedures met guidelines of the National Institutes of Health Guide for the Care and Use of Laboratory Animals (NIH Publication number 85–23, revised 2011) and were approved by the Institutional Animal Care and Use Committee of Grand Valley State University (Protocol number: 13-01-A).

### Drugs and injections

Norbinaltorphimine (nor-BNI; Tocris Biosciences, Ellisville, MO) was dissolved in 0.9% saline solution for intraperitoneal (i.p.) injections.

### Ethanol liquid diet

Rats were fed a nutritionally complete liquid diet composed of 10% ethanol, a commercially available liquid nutritional supplement, vitamins, and minerals for 28–30 days [23, 24]. Control animals received a similar diet with sucrose isocalorically substituted for ethanol. A fresh diet was be prepared daily and given to the animals at the onset of their dark cycle. Animals in the ethanol condition received unlimited access to the diet, whereas animals in the control group received an amount equal to the average intake of the ethanol group on the previous day, in order to control for differences in caloric intake. This procedure has been previously shown to produce pharmacologically relevant blood alcohol levels (BALs) and induce physical signs of dependence [23].

### Forced swim test

The forced swim test is a two-day procedure in which rats swim under conditions in which escape is not possible [25]. On the first day of the forced swim test, rats were placed in a clear, 65-cm-tall by 25-cm-diameter cylinder filled to 48 cm with 25°C water for 10 minutes, where they eventually adopted a posture of immobility in which they made only the movements necessary to keep their heads above water. 24 hours later, rats were retested for 5 minutes under identical swim conditions. Retest sessions were video recorded and scored by raters unaware of the treatment condition. The behavior measured was total immobility time, defined as floating to maintain the head above water with only minor paw movement.

### Experiment 1

Experiment 1 sought to examine the effects of nor-BNI on immobility in the forced swim test during acute withdrawal from ethanol. Rats (n = 35) were fed an ethanol or control liquid diet for 28–30 days as described above. 24 hours after removal of the diet, rats were exposed to 10 minutes of forced swim stress. Animals were injected with nor-BNI (20 mg/kg, i.p.) or saline immediately following this procedure, and were then tested in the forced swim test 24 hours later for 5 minutes. A 20 mg/kg dose of nor-BNI was used in these experiments because previous work in our laboratory has shown that this dose has the ability to reduce anxiety-like behaviors following withdrawal in rats exposed to chronic ethanol under similar conditions [9, 15]. A pretreatment time of 24 hours was chosen because previous research has shown that nor-BNI is most selective for KORs at 24 hours after administration as opposed to earlier times [26].

### Experiment 2

Experiment 2 examined the ability of nor-BNI to reverse immobility in the forced swim test in rats with a history of ethanol dependence following a protracted period of abstinence. A separate group of rats (n = 33) were fed an ethanol or control liquid diet for 28–30 days as described. The diet was then removed, and rats were not subjected to any experimental procedures for three weeks. However, routine husbandry was performed, and rats were weighed and observed for any physical or behavioral abnormalities (ex. weight loss of greater than 20%, inappetence, weakness, moribund state, central nervous system dysfunction) daily. At the conclusion of this three-week period, rats were exposed to 10 minutes of forced swim stress. Similar to Experiment 1, rats were injected with nor-BNI (20 mg/kg, i.p.) or saline immediately following this procedure, and were then tested in the forced swim test 24 hours later for 5 minutes.

### Data analysis

Data for these experiments were analyzed using a three-way analysis of variance (ANOVA) with withdrawal condition, diet, and nor-BNI dose as between-subjects factors. Tukey’s test was used for post hoc analysis as warranted.

## Results

Rats tested during acute withdrawal given the ethanol liquid diet consumed an average of 10.04 g/kg of alcohol per day (SD = 3.12, range = 1.64–28.70) over the course of the liquid diet administration. These levels of ethanol consumption have previously been shown to induce pharmacologically relevant BALs, which induce physical symptoms of dependence and withdrawal [23]. Although the average intake per animal was determined by the total consumption of fluid per cage, the average body weights of each animal did not significantly differ from each other, suggesting that significant differences in the amount of fluid intake between animals housed together are not likely. In addition, no significant differences were observed in body weight between animals exposed to the ethanol diet versus similarly housed animals exposed to a control diet (ethanol mean = 278.53, SD = 13.67; control mean = 291.87, SD = 25.92).

Similar to the acute withdrawal condition, rats tested during protracted abstinence consumed pharmacologically relevant levels of ethanol when fed the ethanol liquid diet (mean = 11.44 g/kg of alcohol per day, SD = 3.29, range = 3.03–25.15). In addition, the average body weights of each animal did not significantly differ from each other, and no significant differences were observed in body weight between animals exposed to the ethanol diet versus similarly housed animals exposed to a control diet (ethanol mean = 262.02, SD = 28.13; control mean = 267.89, SD = 33.38).

**Fig 1:**
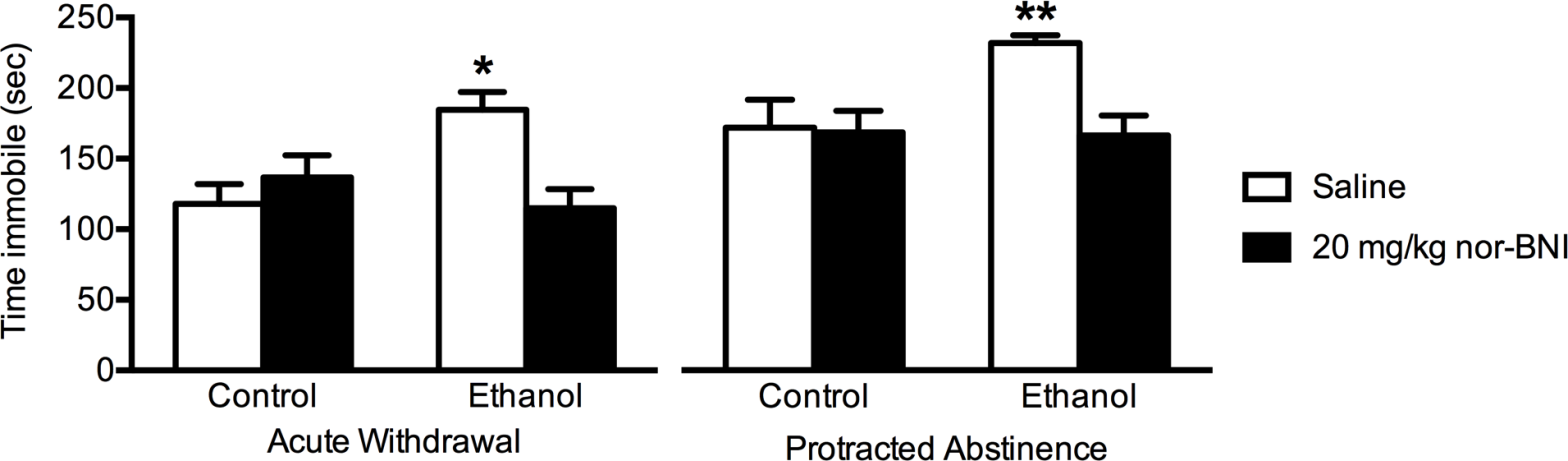
Attenuation of increased immobility during in the forced swim test by nor-BNI in ethanol-dependent rats during acute withdrawal and protracted abstinence. Rats (n = 6-9/group) were exposed to an ethanol or control liquid diet for 28–30 days. In the acute withdrawal condition, rats were exposed to 10 minutes of forced swim stress 24 hours after removal of the diet and then injected with nor-BNI (20 mg/kg, i.p.) or saline immediately following this procedure. Rats were then tested in the forced swim test 24 hours later for 5 minutes. In the protracted abstinence condition, rats were left undisturbed for 3 weeks after removal of the liquid diet. At the end of this period, rats were exposed to 10 minutes of forced swim stress and then injected with nor-BNI (20 mg/kg, i.p.) or saline in a similar manner to the acute withdrawal condition. 24 hours later, rats were examined in the forced swim test for 5 minutes. Data are expressed as mean + SEM. *p < 0.05 compared to all other groups in the acute withdrawal condition, Tukey’s test. **p < 0.05 compared to all other groups in the protracted abstinence condition, Tukey’s test.

There was a significant interaction between liquid diet condition and antagonist treatment on the amount of time spent immobile in the forced swim test (F(1,68) = 12.64, p < 0.001, Fig 1). Further analysis demonstrated that ethanol-dependent rats spent more time immobile compared to controls, an effect that was reversed by injection with nor-BNI during both acute withdrawal and protracted abstinence (p < 0.05, Tukey’s test). There was also a significant main effect of withdrawal condition on time immobile (F(1,68) = 19.18, p < 0.0001). Rats tested during protracted abstinence from ethanol showed more immobility compared to those examined during acute withdrawal across all conditions.

## Discussion

The findings of this study are consistent with previous data demonstrating that animals exposed to chronic ethanol demonstrate long-term behavioral changes in models of depression [10, 11]. Furthermore, the current experiments found that the KOR antagonist nor-BNI reversed these behavioral changes in rats during acute withdrawal and protracted abstinence from ethanol. These results suggest that the DYN/KOR system is involved in regulating long-term behavioral changes associated with ethanol dependence.

Previous research has shown that stress-related behaviors associated with withdrawal from ethanol tend to diminish over time [7]. In contrast, rats examined in the present study spent an overall greater amount of time immobile following protracted abstinence compared to rats tested during acute withdrawal. One possible explanation for this finding is that certain types of behavioral changes associated with ethanol withdrawal may be differentially affected by the duration of abstinence. For example, rats exposed to chronic ethanol vapor show a greater enhancement of ethanol self-administration when tested 2 hours following ethanol vapor exposure compared to when examined after 2–5 weeks of abstinence [7]. Additionally, ethanol-exposed rats spend less time exploring the open arms of the elevated plus maze, an indication of a heightened anxiety-like state, during acute withdrawal compared to a protracted period of abstinence [7]. In contrast, when examined in the forced swim test, rats exposed to chronic ethanol vapor show increased immobility when compared to controls during protracted abstinence, but not during acute withdrawal [10]. Taken together, these data suggest that while motivational and anxiety-like behaviors may diminish with time during abstinence, depressive-like behaviors may be enhanced. Additional research examining the underlying mechanisms for this difference would provide further insight into this issue.

Our study found that rats exposed to chronic ethanol showed increased immobility in the forced swim test when examined during acute withdrawal and protracted abstinence from ethanol. However, other studies comparing depressive-like behavior at similar time points have only found increases in immobility during protracted withdrawal [10, 27, 28]. The reason for this discrepancy is unclear, but one explanation may be the method used to induce ethanol dependence. Our experiments used an ethanol liquid diet procedure in which rats were fed a nutritionally complete liquid diet in place of standard laboratory chow. In these previous studies, animals were exposed to chronic ethanol vapor [10], extended two-bottle ethanol choice access [27], or ethanol injections [28], while still receiving access to standard food and water. Although the ethanol liquid diet is a well-established method for ethanol dependence induction [8, 9, 23, 24], previous research has shown that changes in diet may influence stress-related behaviors [29, 30]. It is possible that the liquid diet acted as a mild stressor itself, contributing to the increase in time immobile during acute withdrawal. However, only ethanol liquid diet fed rats showed an increase in immobility, and the level of immobility in rats fed the control diet was similar to that previously seen in air-exposed controls using the ethanol vapor procedure [10], suggesting that the change in diet did not significantly alter behavior.

KOR antagonism has previously been shown to have general antidepressant-like effects in the forced swim test [31–33]. In contrast, our study found that nor-BNI only reduced immobility in ethanol dependent rats, and did not display antidepressant-like effects in controls. One possible explanation for this discrepancy is that control rats had a lower baseline level of immobility, resulting in a floor effect. A previous study found that nor-BNI reduced immobile counts in Wistar Kyoto rats, which had high baseline levels of immobility, but did not reduce the number of immobile counts in Sprague Dawley rats, which displayed a lower overall immobility baseline [33]. Our results are consistent with this previous finding, as nor-BNI only showed antidepressant-like effects in the ethanol diet-fed rats, which displayed greater levels of immobility compared to controls.

One issue to consider is that BALs were not directly measured in the animals tested, which could possibly lead to wide variations in BALs between animals. However, as stated in the results, body weights between animals did not significantly differ from one another, suggesting that animals housed within groups consumed a similar amount of the liquid diet and subsequently, similar levels of ethanol. Previous work in which rats were fed a similar ethanol liquid diet showed that this procedure leads to BALs in the range of 100–150 mg% [23, 34, 35]. This level of intake has been shown to produce significant signs of physical withdrawal [23, 34] and long-term alterations in the function of biological stress systems [34]. Our laboratory has also demonstrated that rats maintained under nearly identical ethanol liquid diet conditions show increases in anxiety-like behavior during acute and protracted withdrawal from ethanol [9, 15]. Finally, ongoing experiments in our laboratory that have measured somatic withdrawal signs using a method described in detail previously [23, 24] found that ethanol-diet fed rats show significantly higher somatic withdrawal scores compared to controls when exposed to the same liquid diet procedure used in the present study (S4 Table). These similarities demonstrate that the liquid diet procedure used was sufficient to induce ethanol dependence.

As previously stated, research has shown that rats will self-administer ethanol for up to eight weeks following chronic exposure to ethanol in the absence of physical withdrawal signs [12]. Furthermore, injections of the KOR antagonist nor-BNI into the central nucleus of the amygdala attenuates ethanol drinking following a protracted period of abstinence without affecting signs of physical withdrawal [13]. This previous work suggests that continued ethanol self-administration is more likely due to negative affective-like states rather than physical withdrawal. The present study demonstrates that behaviors indicative of depressive-like states can persist beyond the acute withdrawal phase, and that this behavioral change is, in part, regulated by the DYN/KOR system. When considered together, these data suggest that the DYN/KOR system is a key mediator of the negative affective-like states that contribute to relapse.

Although the present study suggests that KORs play a prominent role in the long-term behavioral changes associated with ethanol withdrawal, it is important to note that other stress-related neurobiological systems may interact with the DYN/KOR system to produce these effects. Previous work has shown that KOR-induced behavioral changes associated with drugs of abuse may be mediated by corticotropin-releasing factor (CRF) and noradrenergic mechanisms [36], and DYN, CRF, and norepinephrine are co-localized in various brain regions [37–41]. Nonetheless, the findings of the current study further support the hypothesis that the DYN/KOR system plays a significant role in regulating behavioral changes associated with negative affective-like states following chronic ethanol exposure. Overall, these findings indicate that long-term alterations in the DYN/KOR system are likely involved in regulating depressive-like states associated with alcohol dependence.

## Supporting information

S1 Table Intake data for ethanol and control liquid diet. (XLSX)

S2 Table Time immobile data in the forced-swim test. (XLSX)

S3 Table Somatic withdrawal observation data. (XLSX)

S4 Table Summary and analysis of somatic withdrawal observation scores. Rats (n = 40) were maintained on an ethanol or control liquid diet for 28–30 days as described in the Methods. To confirm physical ethanol dependence, rats were rated for somatic withdrawal signs. 24 hours after removal of the diets, rats were placed individually in a Plexiglas cage with a bed of wood shavings for 5 minutes. Sessions were video recorded and rats were rated by trained observers for the presence of a ventromedial distal limb flexation response, tail stiffness, and abnormal body posture. Each sign was rated on a 0 (absent) to 2 (severe) scale. Rats fed an ethanol liquid diet had significantly higher somatic withdrawal signs compared to controls (t(38) = 4.74, p < 0.0001.
(PDF)
