# Supplementary material for: Kappa opioid regulation of depressive-like behavior during acute withdrawal and protracted abstinence from ethanol
Source: PLoS One. 2018 Sep 28;13(9):e0205016. doi: 10.1371/journal.pone.0205016 (PMC6161915; doi:10.1371/journal.pone.0205016)
Supplement: S4 Table — Rats (n = 40) were maintained on an ethanol or control liquid diet for 28–30 days as described in the Methods. To confirm physical ethanol dependence, rats were rated for somatic withdrawal signs. 24 hours after removal of the diets, rats were placed individually in a Plexiglas cage with a bed of wood shavings for 5 minutes. Sessions were video recorded and rats were rated by trained observers for the presence of a ventromedial distal limb flexation response, tail stiffness, and abnormal body posture. Each sign was rated on a 0 (absent) to 2 (severe) scale. Rats fed an ethanol liquid diet had significantly higher somatic withdrawal signs compared to controls (t(38) = 4.74, p < 0.0001. (PDF) [file pone.0205016.s004.pdf]

Unpaired t test with equal SD		
1	Table Analyzed	Withdrawal Scores
2		
3	Column B	Ethanol
4	vs.	vs.
5	Column A	Control
6		
7	Unpaired t test	
8	P value	< 0.0001
9	P value summary	****
10	Significantly different? (P < 0.05)	Yes
11	One- or two-tailed P value?	Two-tailed
12	t, df	t=4.744 df=38
13		
14	How big is the difference?	
15	Mean ± SEM of column A	1.450 ± 0.2112, n=20
16	Mean ± SEM of column B	3.250 ± 0.3152, n=20
17	Difference between means	1.800 ± 0.3794
18	95% confidence interval	1.032 to 2.568
19	R squared	0.3720
20		
21	F test to compare variances	
22	F,DFn, Dfd	2.227, 19, 19
23	P value	0.0891
24	P value summary	ns
25	Significantly different? (P < 0.05)	No
